# Combination prosthetic design providing a superior retention for mid-facial defect rehabilitation: A Case Report

**DOI:** 10.4317/jced.53513

**Published:** 2017-04-01

**Authors:** Supassra Nilanonth, Prana Shakya, Natdhanai Chotprasert, Theerathavaj Srithavaj

**Affiliations:** 1 DDS, Grad Dip, Maxillofacial prosthetic service, Faculty of Dentistry, Mahidol University. Bangkok, Thailand; 2 BDS, MSc, Teaching assistant, Maxillofacial Prosthetic Service, Faculty of Dentistry, Mahidol University. Bangkok Thailand; 3 DDS, MSc, PhD, Lecturer, Maxillofacial Prosthetic Service, Faculty of Dentistry, Mahidol University. Bangkok Thailand; 4 DDS, MS, Assistant Professor, Maxillofacial Prosthetic Service, Faculty of Dentistry, Mahidol University. Bangkok Thailand

## Abstract

Large maxillofacial defects from malignant tumor treatment are rarely rehabilitated by surgical reconstruction alone. Ameloblastic carcinoma, a rare aggressive odontogenic malignant tumor, requires wide surgical excision to gain a tumor-free margin. In the post-surgical defect, prosthetic rehabilitation is the treatment of choice to restore function and esthetics. Moreover, an intra-oral prosthesis such as an obturator restores speech, mastication and deglutition. Retention of the obturator is a major problem while rehabilitating large defects. The existing anatomical structures from the defect with the help of magnet attachments are suitable to enhance retention, stability and support of the prostheses. This case report presents a patient with an intraoral and extra-oral combination defect following surgical resection of ameloblastic carcinoma and describes the prosthetic techniques and design considerations for a magnet-retained obturator and mid-facial prosthesis. An implant-retained mid-facial prosthesis was fabricated. The retention of combined prostheses was obtained from the remaining right posterior teeth only. The patient had an unfavorable defect due to the large size and presence of scar contracture that vertically tends to dislodge the obturator. Magnet attachments were used to combine the facial and oral prosthesis, minimize the vertical dislodging forces and enhance retention. In addition, the retention was also gained from the scar band at lower border of mid-facial defect that avoided the need for more implants surgery. Magnet attachment with anatomical structure of the mid-facial defect provides an acceptable means of retention in large extraoral-intraoral combinations defects, improving the function, esthetic and the patients’ quality of life.

** Key words:**Mid-facial prosthesis, obturator, magnet attachment, maxillectomy.

## Introduction

Lateral mid-facial defects from malignant tumor treatment are rarely rehabilitated by surgical reconstruction alone. Ameloblastic carcinoma, a rare aggressive odontogenic malignant tumor, requires wide surgical excision to gain tumor-free margin, resulting in a large defect, which might involve parts of the face (1,2). Therefore, rehabilitation with maxillary obturator and facial prosthesis are the treatment of choice to restore speech, deglutition, and facial appearance improving patient’s quality of life (3).

Mid-facial defects are defects located in the middle third of the face in horizontal plane with intraoral communication. These defects are classified as: midline mid-facial defects, which include the nose and/or upper lip, and lateral midfacial defects that include the cheek and orbit contents. Combinations of these two categories also exist (4). Midfacial defects involving the maxilla, hard palate, and paranasal sinuses require intra-oral and extra-oral prostheses for rehabilitation. Post-surgical outcomes of combination defects are often prosthetically unfavorable (1,5) due to the location of the defects, deficiency of remaining hard and soft tissues, inadequate number and alignment of abutment teeth, and quality of existing soft tissue. The use of magnet attachment to enhance retention, stability, and support for combination of obturator and facial prosthesis in large defects can be considered (6).

The use of craniofacial implant for prosthetic rehabilitation is a useful and predictable treatment modality. However, the survival rate of craniofacial implant varies in sites depending on bone quantity and quality, hygienic care, soft tissue thickness and radiotherapy (7). The success rates of craniofacial implants have been reported to be 0% to 33% at the glabella area, 65% to 70% in superior and lateral orbital site, and 94.1% in temporal bone site. The lower success rate might be because of the bone at orbital rim has limited volume with dense cortical bone and glabella area has high density but poor vascular supply (8,9). The reported success rate of craniofacial implant was influential in the failure of two implants at the glabella and superior orbital rim sites. Only one implant in the temporal bone site remained. This clinical report presents an alternative approach to combine facial and obturator prostheses with magnet attachment.

## Case Report

A 56 year-old female patient was referred for definitive prosthetic rehabilitation with a chief complaint of poor facial appearance and loss of masticatory function and speech. The patient had a past medical history of ameloblastoma in 2005 and had subtotal left maxillectomy. A definitive obturator was delivered. A new mass was detected in 2007 and diagnosed as ameloblastic carcinoma. A wide excision for tumor-free margin was extended to lateral wall of maxillary sinus. Recurrent ameloblastic carcinoma was again detected in 2009 and had total left maxillectomy, left orbital exenteration, omohyoid neck dissection, and reconstruction with radial forearm free-flap. After which, patient has been re-evaluated every 6 months, the defect was clear from recurrent tumor.

**Figure 1:**
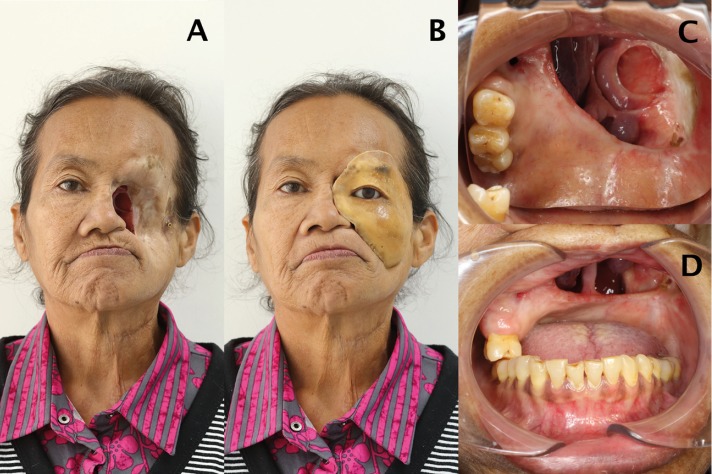
A) Mid-facial defect, B) deteriorated facial prosthesis, C) Maxillary defect; occlusal view and D) frontal view.

Initially, the patient had a facial prosthesis retained by three extraoral implants. After three years, two implants failed as a result of inadequate hygiene maintenance causing peri-implantitis. One extra-oral implant at the zygomatic process of temporal bone remained. Clinical examination revealed a left lateral midfacial defect. Intraoral defect remains with a small residual maxillary tuberosity on left side along with right posterior teeth, which classified as Aramany class IV maxillary defect (10) (Fig. 1).

The definitive obturator with a linear design was fabricated where retention was obtained from the remaining teeth only. Due to the mucosal epithelial lining without a skin graft, it was unable to gain retention from the lateral wall. One remaining implant cannot provide adequate retention to retain midfacial prosthesis due to the large size of the defect. Therefore, in this clinical report, magnets were used to combine unfavorable retention of the facial prosthesis and obturator (6,11).

**Figure 2:**
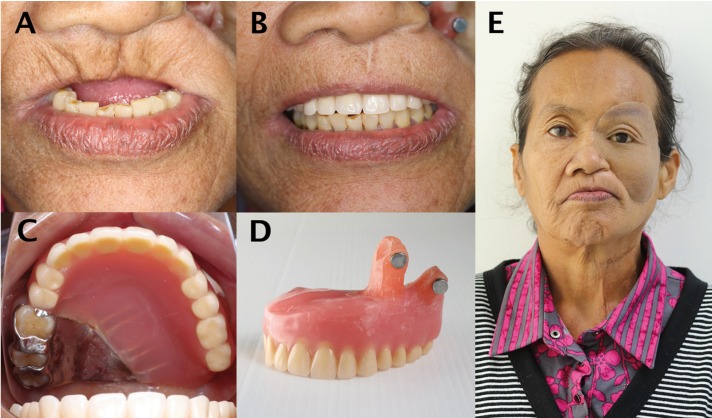
Extraoral view showed lip support from the obturator: A) without obturator, B) with obturator, C) intraoral view, D) the superior extension with metal keeper done, and E) the patient with new facial prosthesis.

For fabrication of the obturator, a linear design was chosen and teeth preparation with 2 embrasures rest on #26, 27, and 28 was done and impression was made with irreversible hydrocolloid (Jeltrate, Densply, York, USA) and were poured with type IV stone for Co-Cr framework fabrication. The framework was tried in and altered casts technique was made by Coe-comfort (GC, Illinois, USA) functional impression. Bite block was tried in, interocclusal bite registered, and casts mounted the casts on a semi-adjustable articulator by using face-bow transfer (HanauTM Springbow, Whip mix, Louisville, USA). Artificial teeth, A 3.5 (Yamahachi Acrylic Resin Teeth, Gamagori City, Japan) was chosen. Final teeth arrangement was completed and verified clinically for lip contour and occlusal plane were evaluated with monoplane occlusion on the defect side. The obturator was processed with heat-cured acrylic resin (Figs. 2A-C).

**Figure 3:**
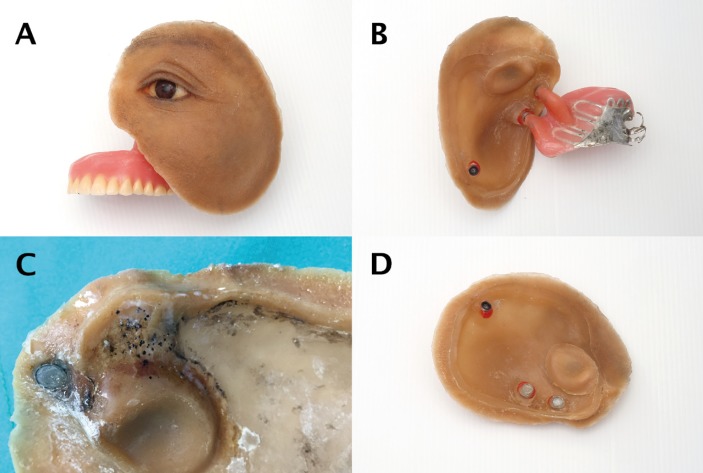
Combined prostheses: A) anterior view, B) posterior view shows the part that is hung over the lower border of scar band to resist vertical and lateral dislodgement, C) Fungal colonization causing silicone deterioration, and D) extended acrylic framework to prevent silicone from moisture.

An impression of the lateral left facial defect was made with addition silicone (Multisil-Epithetik, Bredent, Senden, Germany) and the impression was picked up with irreversible hydrocolloid impression material and poured with type IV stone for master cast. The ocular prosthesis was fabricated by matching color from the companion eye with 3D ocular prosthesis technique. (Thai letter patent No.36414) The wax-pattern was fabricated and the ocular prosthesis was positioned by comparing to the right side of the face and 3 dimensional orientation of the iris position was determined. The facial wax pattern was completely carved and the characterization of skin details was created, including wrinkles, grooves, and surface texture on the facial surface, and the facial wax pattern was tried on the patient. The wax pattern on the intaglio surface was reduced to allow thickness of approximately 6-7 mm and the final wax pattern was sealed to the cast. Type IV stone was poured onto the facial surface of the lower mold for fabricating the upper mold. Acrylic framework was fabricated onto the lower mold with auto-polymerized clear acrylic resin with 2-3 mm in thickness. The extended acrylic framework prevented the silicone from direct contact with the moisture from the oro-nasal cavity (Figs. 3C,D). The platinum cured primer was applied to enhance the bond of acrylic framework and silicone (A 304, Factor II, Arizona, USA). The prosthesis was processed using medical grade silicone (MDX4-4210, Dow Corning Corp, Michigan, USA) with intrinsic staining colors (Functional Intrinsic II, Factor II, Arizona, USA) comparing to the adjacent skin of the defect. After 3 days of full curing at room temperature, the molds were separated and clinically tried for final confirmation of the prosthesis position.

Two lab analogues (Maxi, Technovent limited, South Wales, UK) were attached to the upper part of the obturator at the surface facing the facial prosthesis using auto-polymerizing acrylic resin (Fig. 2D). The acrylic resin extension connecting the intraoral and extraoral prosthesis is hung at lower part of scar band (Fig. 3B). The housing part of 2 magnet attachments (Maxi magnet, Technovent limited, South Wales, UK) and 1 locator male processing (Zest Anchors, Escodido, CA, USA) was embedded to au-to-polymerized clear acrylic resin, attached to 2 lab analogues and 1 locator abutment, and splinted 3 attachments with au-to-polymerized acrylic resin for securing the position. Picked up for fixing the splinted attachment with auto-polymerized clear acrylic resin, the acrylic bands were cut when the position of attachments was stabilized. The facial prosthesis was secured in place with the retention from 3 attachments (Fig. 2E). Extrinsic staining with extrinsic colors (Dry Pigment, Factor II, Arizona, USA) was done to match the adjacent skin tone (Fig. 3A). Medical adhesive (A-564-2 Silicone Adhesive, Factor II, Arizona, USA) was applied on the facial surface of the facial prosthesis and the silicone-acrylic junction for stabilizing the color and increasing the tear resistance of the prosthesis margin.

## Discussion

Large maxillofacial defects that have oronasofacial communications require intraoral and extraoral prosthesis for rehabilitation. However, large communication defects are not favorable because they affect the size and weight of the prostheses and they have limited retention that can be gained from the anatomical structure of the defect. Retention of the prostheses can be provided from anatomic undercuts, attachments, medical adhesive and implants (12,13). In this case, we designed two prostheses that were combined by magnet attachments to enhance the retention and stability of both the extraoral and intraoral prostheses. The acrylic extension at superiolateral part of the obturator was designed to hang at the lower border of scar band. This design enabled the both the obturator and the extraoral prosthesis to gain retention from each other and resist vertical dislodgement force, thereby contributing in its retention and stability. Furthermore, the palatal bulb of the obturator designed to hang at the scar band of the extraoral defect reduced the cantilever effect, which helped in preservation of the periodontal health of the remaining abutment teeth. The design using two magnets and one locator attachments provided adequate retention for the facial prosthesis without using medical adhesive and reduced the silicone-skin contact area, which prevents the risk of physical irritant contact dermatitis (14). In addition, the lightweight combination prostheses was gained by fabricating a bulbless obturator 

acrylic resin and the reduction of facial silicone thickness. Magnet attachments not only provide the retention for the facial prosthesis but also enable the patient to self-align the prosthesis in the exact position with ease.

Fungal colonization that is commonly reported in silicone prostheses is caused by moisture, warmth, and silicone surface porosity. In addition, this condition causes permanent black discoloration and silicone deterioration (14,15). In our design, we aim to reduce the fungal colonization by extending the acrylic framework underneath the facial silicone to cover the part of nasal cavity, and thereby prevent the direct exposure of the silicone to the moisture from respiration and body temperature.

## Conclusions

Retention of the prosthesis is a major problem while rehabilitating large defects. To obtain optimal retention, stability, and support of prostheses, magnet attachments were used for interconnection of intraoral-extraoral prostheses as shown in our alternative painless approach. This properly designed combined prosthesis can significantly reduce weight and enhance retention and stability with ease for patients and no more surgery.
